# Oral (−)-Epicatechin Inhibits Progressive Tau Pathology in rTg4510 Mice Independent of Direct Actions at GSK3β

**DOI:** 10.3389/fnins.2021.697319

**Published:** 2021-06-16

**Authors:** Katriona L. Hole, Lydia E. Staniaszek, Gayathri Menon Balan, Jody M. Mason, Jon T. Brown, Robert J. Williams

**Affiliations:** ^1^Department of Biology and Biochemistry, University of Bath, Bath, United Kingdom; ^2^Institute of Biomedical and Clinical Sciences, University of Exeter Medical School, Exeter, United Kingdom

**Keywords:** Alzheimer’s disease, Tau & phospho-Tau protein, flavonoid, glycogen synthase kinase 3 β, dementia, rTg4510 mouse, Epicatechin, polyphenol

## Abstract

Aggregation of the microtubule-associated protein tau into paired helical filaments (PHFs) and neurofibrillary tangles is a defining characteristic of Alzheimer’s Disease. Various plant polyphenols disrupt tau aggregation *in vitro* but display poor bioavailability and low potency, challenging their therapeutic translation. We previously reported that oral administration of the flavonoid (−)-epicatechin (EC) reduced Amyloid-β (Aβ) plaque pathology in APP/PS1 transgenic mice. Here, we investigated whether EC impacts on tau pathology, independent of actions on Aβ, using rTg4510 mice expressing P301L mutant tau. 4 and 6.5 months old rTg4510 mice received EC (∼18 mg/day) or vehicle (ethanol) via drinking water for 21 days and the levels of total and phosphorylated tau were assessed. At 4 months, tau appeared as two bands of ∼55 kDa, phosphorylated at Ser262 and Ser396 and was unaffected by exposure to EC. At 6.5 months an additional higher molecular weight form of tau was detected at ∼64 kDa which was phosphorylated at Ser262, Ser396 and additionally at the AT8 sites, indicative of the presence of PHFs. EC consumption reduced the levels of the ∼64 kDa tau species and inhibited phosphorylation at Ser262 and AT8 phosphoepitopes. Regulation of the key tau kinase glycogen synthase kinase 3β (GSK3β) by phosphorylation at Ser9 was not altered by exposure to EC in mice or primary neurons. Furthermore, EC did not significantly inhibit GSK3β activity at physiologically-relevant concentrations in a cell free assay. Therefore, a 21-day intervention with EC inhibits or reverses the development of tau pathology in rTg4510 mice independently of direct inhibition of GSK3β.

## Introduction

Currently, there is no preventative treatment strategy for Alzheimer’s disease (AD), the most common cause of dementia. A well-tolerated intervention is urgently needed: one that can target multiple aspects of the heterogenous disease pathology and can be delivered at a population level, throughout adult life. Given this, there is considerable interest in lifestyle interventions based around polyphenol-rich diets, which have been shown to improve cognition and reduce AD biomarker burden (van de Rest et al., 2015; Hill et al., 2019).

Alzheimer’s disease is a neurodegenerative disease characterized by the deposition of amyloid plaques and neurofibrillary tangles (NFTs) in the brain, formed by aggregation of Amyloid-β (Aβ) peptides and tau, respectively. Around 15–20 years prior to symptom onset Aβ begins to accumulate, activating downstream pathological cascades and triggering the development of tau pathology. Aβ has been at the forefront of drug development since the inception of this amyloid cascade hypothesis. However, Aβ-targeting drugs continue to disappoint at clinical trials probably because they are being delivered too late in the disease process, when Aβ-initiated pathways are well underway and significant synaptic and neuronal damage has already occurred (Gómez-Isla et al., 1996; Long and Holtzman, 2019).

The microtubule associated protein tau may be a more tractable target, as tau pathology typically appears at a later stage and correlates much better with neuronal loss and cognitive deterioration than Aβ (Giannakopoulos et al., 2003; Nelson et al., 2012). Physiologically, tau is a natively unfolded, soluble protein showing little tendency for aggregation (Mukrasch et al., 2009). However, in AD, tau is hyperphosphorylated by several kinases including glycogen synthase kinase-3β (GSK3β), MAP kinases and CDK5 (Noble et al., 2013). Hyperphosphorylated tau misfolds, leading to the formation of toxic oligomeric tau species which trigger neuronal dysfunction and seed self-propagation (Gerson et al., 2013; Noble et al., 2013; Wang and Mandelkow, 2016). Blocking tau phosphorylation, aggregation and propagation thus has the potential to provide additional disease-modifying benefit beyond direct targeting of Aβ pathology.

The polyphenol (−)-Epicatechin (EC) is a dietary flavonoid of the flavan-3-ol subgroup, found in relatively high concentrations as a monomer in cocoa beans and more widely distributed in oligomeric form as a proanthocyanidin. EC has well characterized signaling actions in neural cells (Schroeter et al., 2007; Bahia et al., 2008) and is a promising intervention for AD due to its low toxicity profile (Ottaviani et al., 2015) and potential for multi-modal targeting (Hole and Williams, 2020). EC has been shown to improve vascular function and cognition in humans (Bernatova, 2018; Haskell-Ramsay et al., 2018), reduce oxidative stress and up-regulate neuroprotective pathways (Schroeter et al., 2001; Spencer et al., 2001; Shah et al., 2010; Rendeiro et al., 2013; Zhang et al., 2016; Navarrete-Yañez et al., 2020). With respect to AD pathology, intervention with EC has been shown to inhibit APP processing in primary cortical neurons (Cox et al., 2015) and reduce Aβ burden in APP^*SWE*^/PS1 mouse models (Zeng et al., 2014; Cox et al., 2015).

There is some evidence to suggest that flavan-3-ol administration could influence tau pathology, as grape seed and lychee extracts reduce tau phosphorylation *in vivo* (Wang et al., 2010; Santa-Maria et al., 2012; Chen et al., 2019), and EC reduced basal tau phosphorylation in aged mice (Navarrete-Yañez et al., 2020). The key tau kinase GSK3β is a promising target for EC as it can increase Akt phosphorylation (Schroeter et al., 2007), which regulates GSK3β activity by inhibitory phosphorylation at Ser9. EC intervention in aged (Navarrete-Yañez et al., 2020) and APP/PS1 (Zhang et al., 2016) mice has been shown to increase phosphorylation of GSK3β via Akt, but this has not been explored in a mouse model of tauopathy.

It is not clear if there is a direct causal relationship between EC administration and the emergence of pathological tau or the extent to which this might be driven by actions at GSK3β. Tau pathology can be modeled in mice using a single point mutant that is sufficient to cause frontotemporal dementia with parkinsonism-17, such as P301L tau (Hutton et al., 1998). rTg4510 mice overexpress htau with the P301L missense mutation and exhibit an age-dependent increase in tau phosphorylation, aggregation, and associated pathology (Ramsden et al., 2005; Santacruz et al., 2005).

Using rTg4510 mice as a model of tauopathy, we sought to determine whether addition of EC to drinking water for 21 days could inhibit tau pathology. Our results suggest that oral intervention with EC reduced the appearance of phosphorylated, high molecular weight forms of tau, but this could not be correlated with any inhibitory actions at GSK3β.

## Methods

### Materials

(−)-Epicatechin (≥90%; E1753) was purchased from Sigma Aldrich for *in vivo* studies. Quercetin dihydrate (≥99%; 1135), (−)-Epicatechin (≥99%; 0977), and Epigallocatechin gallate (EGCG; ≥98%; 0981) were purchased from Extrasynthese for *in vitro* studies. All culture reagents and media were purchased from Gibco. Primary and secondary antibodies as stated in Table 1.

**TABLE 1:** Antibodies used for immunoblotting.

Antibody name	Company and product number
Tau46	Cell Signaling Technology (#4019)
Tau5	Abcam (ab80579)
Phospho-Tau (Ser396; PHF13)	Cell Signaling Technology (#9632)
p-Tau (Ser262)	Santa Cruz Biotechnology (sc-101813)
Anti-Human Phospho-PHF-tau pSer202/Thr205 (AT8)	Invitrogen Antibodies (MN1020)
pGSK3β (Ser9)	Cell Signaling Technology (#9336)
GSK3β (27C10)	Cell Signaling Technology (#9315)
β-Actin	Santa Cruz Biotechnology (sc-47778)
Goat anti-Rabbit IgG, Peroxidase Conjugated	Sigma-Aldrich (AP132P)
Goat anti-Mouse IgG Antibody, Peroxidase Conjugated, H + L	Sigma-Aldrich (AP124P)

### Animals

All procedures were carried out in accordance with the United Kingdom Animal (Scientific Procedures) Act 1986 and were approved by the Universities of Exeter (PPL P29FAC36A) and Bath Animal Welfare and Ethical Review Body.

rTg4510 mice were gifted from Eli Lilly. The rTg(tet-o-TauP301L)4510 mouse model (Ramsden et al., 2005; Santacruz et al., 2005) was bred on a mixed FVB/NCrl + 129S6/SvEvTa background and delivered to the University of Exeter via Envigo (Loughborough, United Kingdom). Male rTg4510 mice were housed on a 12-h light/dark cycle with *ad libitum* access to food and water. rTg4510 mice overexpress the human four-repeat tau gene containing the P301L mutation that has been linked with familial frontotemporal dementia. Transgenic gene expression is under the control of the Ca^2+^-calmodulin kinase II promoter and can be repressed with doxycycline.

### EC Dosing Regime

Four month (*n* = 15 for each group) and 6.5 month (*n* = 10 for each group) old, male mice were administered EC (3 mg/ml) or vehicle (0.1% ethanol v/v) in their water supply for 21 days prior to sacrifice. Average intake of EC per day was 17.27 ± 1.20 mg/mouse/day (SEM) for the 4-month group and 19.03 ± 2.54 mg/mouse/day (SEM) for the 6.5-month group. There were no differences in drinking volumes between the EC and vehicle only groups. At the end of the treatment phase, animals were sacrificed and the brains rapidly removed and snap frozen on dry ice before processing for biochemistry.

### Brain Processing

Mouse brain was homogenized in 50 mM Tris, pH 8.0, 274 mM NaCl, 5 mM KCl, 2 mM EGTA, 2 mM EDTA, and cOmplete^TM^, EDTA-free protease inhibitor cocktail. Homogenates were centrifuged at 15,000 × *g* for 15 min, and the supernatant was collected as a total soluble fraction for immunoblotting. Protein concentrations were determined using the Bradford assay (BioRad).

### Primary Neuron Culture

Primary cortical neuronal cultures were prepared as described previously (Law et al., 2018). Cortices were dissected from embryonic day 15 CD1 mouse embryos and mechanically dissociated using a fire-polished Pasteur pipette, coated in heat inactivated Fetal Bovine Serum, in PBS-Glucose [6 mM D-glucose (Sigma), Ca^2+^ and Mg^2+^ free]. Neurons were plated into 6 well tissue culture plates (Nunc) that were precoated with 20 mg/mL poly-D-lysine (Sigma). Neurons were maintained in Neurobasal medium minus phenol red, supplemented with B-27, 2 mM glutamine, 100 mg/mL streptomycin, and 50 mg/mL penicillin (Invitrogen), at 37°C in a humidified atmosphere of 95% air and 5% CO2.

### EC Treatment of Primary Cortical Neurons

Stock EC was prepared in 0.5% acetic acid (v/v) in ethanol:H_2_O (8:2; v/v). The media from DIV14 primary cortical neurons was replaced with conditioned media prior to treatment with EC or vehicle for 15 min. Neurons were lysed in radioimmunoprecipitation buffer [150 Mm NaCl, 25 mM Tris, 0.5% Sodium deoxycholate, 0.1% SDS, 1% Non-idet P-40, pH 7.4 made complete with 2 mM EDTA (pH 8), cOmplete^TM^, EDTA-free protease inhibitor cocktail, PhosSTOP^TM^ phosphatase inhibitor] and detached by cell scraping. Lysates were centrifuged at 20,000 × *g* for 20 min at 4°C and the supernatant retained. Samples were diluted 1:4 in Laemmli sample buffer (10% 2-mercaptoethanol) and boiled for 2 min.

### Immunoblotting

Samples were resolved by 10% (GSK3β detection) or 12% (Tau detection) Tris–glycine SDS-PAGE before transfer to 0.45 μm nitrocellulose membrane (GE Healthcare). Following transfer, the membranes were blocked in TBS + 5% milk for 30 min at rtp. Membranes were washed briefly with TBS-T before incubation with the primary antibodies (1:1000) in TBS-T + 1% milk overnight at 4°C. Membranes were then washed with TBS-T and incubated with secondary antibodies (1:2500) in TBS-T + 1% milk for 1 h at rtp. Following washing with TBS-T and then TBS, bound antibodies were detected using Amersham ECL^TM^ Western Blotting Detection Reagent (GE Healthcare). Western blots were imaged and quantified using the Fusion-SL Chemiluminescence System (Vilber Lourmat).

Primary antibodies used for immunoblotting (Table 1). Anti-Mouse IgG Antibody, (H + L) HRP conjugate and Anti-Rabbit IgG Antibody, HRP conjugate secondary antibodies (1:2500; Millipore), were used as in accordance with the source species.

### Luciferase Assay

Glycogen synthase kinase 3β Kinase Enzyme System was used with the ADP-Glo^TM^ assay (Promega) in accordance with manufacturers guidelines. This assay utilizes recombinant full-length human GSK3β, a GSK3 substrate [YRRAAVPPSPSLSRHSSPHQ(pS)EDEEE], derived from human muscle glycogen synthase and a luminescence detection system. Final assay reagent concentrations: 4 μM ATP, 0.04 ng/μL GSK-3β, 20 μg/mL glycogen synthase (substrate). For concentration-response analysis, flavonoids were initially dissolved in 100% dimethyl sulfoxide (DMSO) at a 1 mM concentration before dilution to working concentrations (5% DMSO). The GloMax^®^-Multi + Detection System (Promega) was used to measure luminescence. 4 independent assays were undertaken for each flavonoid.

### Statistical Analysis

Data were analyzed using GraphPad Prism software (Version 8). Immunoblotting data were analyzed by two-way ANOVA with Bonferroni’s Multiple Comparison Test. GSK3β luciferase assay data were analyzed by one-way ANOVA with Bonferroni’s Multiple Comparison Test.

## Results

### Short Term Intervention With (−)-Epicatechin to rTg4510 Mice Inhibits Tau Phosphorylation

**FIGURE 1:**
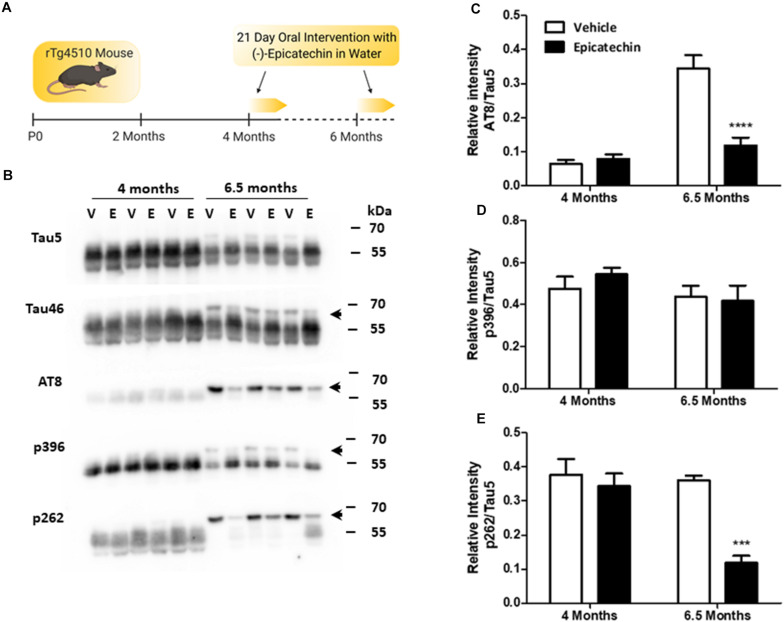
(−)-Epicatechin inhibits age-dependent tau phosphorylation in rTg4510 mice. **(A)** Schematic summarizing the (−)-epicatechin (EC) dosing regime. rTg4510 male mice at 4 and 6.5 months of age were given free access to drinking water supplemented with either EC (3 mg/ml) or vehicle (0.1% ethanol) for 21 days. **(B)** Immunoblots of whole brain homogenates (20 μg) from 4 and 6.5 month old mice exposed to either vehicle (V) or EC (E) probed with antibodies against full length Tau (Tau 5 and Tau 46), the dual phosphorylation sites Ser202/Thr205 (AT8), phosphorylation at Ser396 (p396), and phosphorylation at Ser262 (p262). The arrow indicates the higher molecular weight band at ∼64 kDa. **(C–E)** Quantification of changes in levels of phosphorylation status as a ratio of total Tau (Tau 5). Vehicle (Clear bars), EC (Black bars). Data presented is mean of relative intensity ± SEM (*n* = 5). ***p < 0.001; ****p < 0.0001. Schematic created with BioRender.com.

Previous studies using the rTg4510 mouse model have shown that between 4 and 6 months there is an increase in hyperphosphorylated, oligomeric tau (Ramsden et al., 2005; Santacruz et al., 2005; Sahara et al., 2012). Additionally, at 5.5 months significant neuronal loss can be observed in both the hippocampus and the cortex (Ramsden et al., 2005; Santacruz et al., 2005). With the aim of considering early disease intervention around these critical age-points, 4 and 6.5-months were chosen to assess whether EC treatment could impact on the development and progression of tau pathology. A schematic of the experimental protocol is shown in Figure 1A.

Western blots probed with Tau5 and Tau46 antibodies to detect total tau showed an age-dependent increase in the appearance of a higher molecular weight (HMW) band of tau at 64 kDa, which was detectable at 6.5 months but not at 4 months (Figure 1B). This HMW band, which was most notable when probed with the Tau46 antibody, has been characterized previously as representing hyperphosphorylated, oligomeric tau (Sahara et al., 2012). EC treatment in 6.5-month old mice decreased the levels of 64 kDa tau (*p* < 0.001), as emphasized by a clear redistribution to the lower molecular weight forms of tau (Figure 1B). EC did not, however, affect overall tau levels at either 4 months or 6.5 months giving us confidence that this change did not result from off-target effects at the doxycycline sensitive tetracycline transactivator. Overall, this initial series of observations strongly suggest that short term oral intervention with EC reduces the levels of oligomeric tau.

Blotting with phosphorylation-state specific antibodies against tau demonstrated high basal levels of p396 and p262 at 4 months, but much lower levels of AT8 (pS202/pT205). EC treatment did not affect phosphorylation of tau in 4-month old mice at any of these sites (Figure 1B). Only tau phosphorylation at the AT8 phosphoepitope was significantly increased between 4 and 6.5 months (*p* < 0.0001; Figures 1C–E), although tau phosphorylated at both AT8 and p262 was redistributed to the HMW band in 6.5-month old mice (Figure 1B). At 6.5 months, EC intervention significantly inhibited tau phosphorylation at AT8 (Ser202/Thr205; *p* < 0.0001) and at p262 (*p* < 0.001) compared with vehicle controls (Figures 1B,C,E). The overall levels of phosphorylation at Ser396 were not affected by EC, although there was a redistribution to the 55 kDa band from the 64 kDa band in line with total tau changes (Figures 1B,D). This suggests that Ser396 phosphorylation is not essential for formation of the 64 kDa species.

### (−)-Epicatechin Does Not Inhibit GSK3β

**FIGURE 2:**
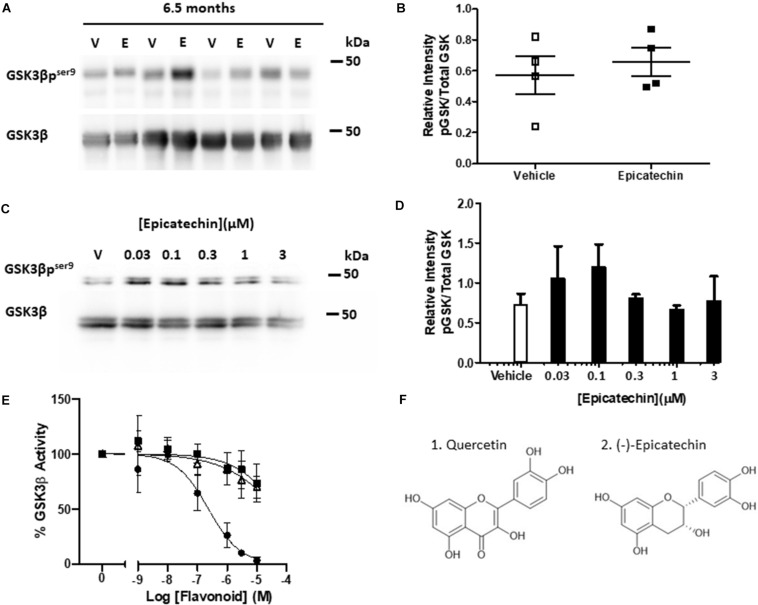
(−)-Epicatechin does not inhibit GSK3β activity either *in vivo* or *in vitro*. **(A)** Immunoblots of whole brain homogenates (20 μg) from 6.5 month-old rTg4510 male mice exposed to either vehicle (V) or EC (E) probed with antibodies against GSK3β and GSK3β phosphorylated at Ser9 (GSK3βp^*ser*9^). **(B)** Scatter plot showing quantification of changes in phosphorylation status of GSK3β as a ratio of total GSK3β for vehicle (open squares) and EC (black squares) treatments, no significant differences (*n* = 4). **(C)** Immunoblots of primary cortical neurons treated for 15 min with vehicle (V) or increasing concentrations of EC (0.03–3 μM) and probed with antibodies against GSK3β and GSK3β phosphorylated at Ser9 (GSK3βp^*ser*9^). **(D)** Quantification of changes in the phosphorylation status of GSK3β as a ratio of total GSK3β. Data is mean ± SEM (*n* = 3). **(E)** Sensitivity of GSK3β catalytic activity to increasing concentrations of flavonoids (1 nM–10 μM). Data presented as% activity related to vehicle control (100%). Quercetin (black circles), EC (black squares), EGCG (open triangles), mean ± SEM (*n* = 4). **(F)** Structures of Quercetin (1) and EC (2).

To investigate whether EC targeted the key tau kinase GSK3β in this model, the levels of pGSK3β were measured at 6.5 months to determine if changes in tau phosphorylation could be correlated with alterations in activity of GSK3β. The levels of pGSK3β were quite variable between the samples but oral intervention with EC did not increase inhibitory GSK3β phosphorylation in rTg4510 mice compared to vehicle controls (Figures 2A,B). To further explore whether acute treatment with EC could increase Ser9 phosphorylation of GSK3β in a system with a less variable background, primary cortical neurons were treated with a range of concentrations of EC known to increase pAkt. No significant increase in GSK3β phosphorylation was measured at any of the concentrations of EC tested (Figures 2C,D).

The dietary flavonoid quercetin, of the flavonol subgroup, (Figure 2F) binds to and inhibits GSK3β directly (Johnson et al., 2011; Carvalho et al., 2017), however, this has not been studied with respect to EC and other flavan-3-ols. To investigate this, we used an *in vitro* luciferase assay to test whether EC and EGCG, which disaggregates tau, inhibit the catalytic activity of human full length GSK3β. Quercetin inhibited GSK3β in a concentration-dependent manner (*p* < 0.0001, IC_50_ = 0.236 μM) with an almost complete loss of activity at the highest concentrations tested (Figure 2E). Both EC (IC_50_ = 54.4 μM) and EGCG (IC_50_ = 51.4 μM) caused a much more modest (∼25%) concentration-dependent inhibition of GSK3β activity (*p* < 0.01), with significant inhibition compared to controls only observed at 10 μM (*p* < 0.05). Neither EC nor EGCG inhibited GSK3β significantly at concentrations that could be considered physiologically relevant (<1 μM).

Therefore, oral administration of EC inhibited tau phosphorylation in the rTg4510 mice and reduced the levels of oligomeric tau independently of either direct or indirect inhibition of GSK3β.

## Discussion

Hyperphosphorylated, oligomeric, soluble tau species are thought to be responsible for tau-driven neuronal toxicity (Spires-Jones et al., 2011) and there is interest in identifying therapeutics that could target these processes. The rTg4510 mouse is a useful model for phenocopying the hyperphosphorylated tau aggregates that are characteristic of human tauopathies, although the accompanying neurodegeneration may be due in part to genomic disruption rather than tau overexpression *per se* so caution is needed when concluding on mechanisms (Gamache et al., 2019). The 64 kDa band of tau found in soluble lysates from rTg4510 mouse brain has been previously well characterized – a result of hyperphosphorylated, oligomeric tau species which correlate better with neuronal loss than Sarkosyl-insoluble tau in this model (Sahara et al., 2012). Our results from 6.5 month old mice showed a reduction of tau at the 64 kDa band, as well as reduced tau phosphorylated at AT8 and Ser262 phosphoepitopes (Figures 1B,C,E). This suggests that short term oral intervention with EC can reduce the levels of these potentially toxic tau species in the rTg4510 mouse.

AT8 and Ser262 are two key phosphorylation sites associated with AD. Phosphorylation at Ser262 inhibits tau binding to microtubules (Biernat et al., 1993) and in a recent proteomics analysis of AD brains, Ser262 phosphorylation was identified as one of the earliest and most distinct tau modifications compared to controls (Wesseling et al., 2020). AT8-targeting antibodies are commonly used to label paired helical filaments (PHFs), and it has been suggested that S202/T205 phosphorylation increases the propensity of tau for aggregation (Rankin et al., 2005). The ability of EC to inhibit phosphorylation at both Ser262 and AT8 is therefore compelling, as it suggests the potential to target early tau pathology and decrease the propensity for tau aggregation. However, further studies in additional tau models will be necessary to confirm this. Interestingly, only phosphorylation at the AT8 phosphoepitope increased with age in these mice (Figures 1C–E). Other studies have shown that phosphorylation at Ser396 and Ser262 residues increase with age in the rTg4510 mouse model (Ramsden et al., 2005; Sahara et al., 2012; Song et al., 2015) possibly reflecting changes in the insoluble fraction which were not studied here.

As GSK3β is a key tau kinase and EC is known to promote activity of its regulator, Akt (Schroeter et al., 2007), we hypothesized that reduced tau phosphorylation could have resulted from EC-induced inhibition of GSK3β. However, EC did not increase inhibitory phosphorylation of GSK3β at Ser9 either *in vivo* (Figures 2A,B) or *in vitro* (Figures 2C,D). This contrasts with reported EC upregulation of the Akt/GSK3β pathway in aged (Navarrete-Yañez et al., 2020) and in APP/PS1 mice (Zhang et al., 2016). The lack of effect in rTg4510 mice could be related in some-way to the use of tauopathy models, as Grape seed polyphenolic extract (GSPE) also failed to increase activity of the Akt/GSK3beta pathway in TMHT tau mice (Wang et al., 2010). However, the tau background would not explain the apparent lack of effect in primary neurons. Although EC induced a predicted bell-shaped concentration response in neurons the signal to noise was insufficient to detect a robust and significant increase in GSKβ S9 phosphorylation.

Reverse screening *in silico* has confirmed GSK3β to be a direct target of quercetin (Carvalho et al., 2017), which, alongside a range of other citrus flavonoids, has been shown to inhibit GSK3β directly (Johnson et al., 2011). This could, therefore, be a possible mechanism for EC inhibition of tau phosphorylation. Indeed, we showed that EC and EGCG dose dependently inhibited GSK3β activity directly (Figure 2E). However, both compounds only inhibited GSK3β activity significantly at micromolar levels which are highly unlikely to be achievable *in vivo* particularly in brain (Wang et al., 2012). Therefore, it can be concluded that direct inhibition of GSK3β by EC is unlikely to be the mechanism by which EC inhibited tau phosphorylation in the rTg4510 mouse model. While GSK3β is a prominent tau kinase, many other kinases contribute to tau phosphorylation (Noble et al., 2013) such as CDK5 (Kimura et al., 2014), microtubule associated protein kinases (Drewes et al., 1995; Gu et al., 2013) and MAPKs (Drewes et al., 1992). Likewise, GSK3β is not the only tau-associated kinase that can be modulated by flavonoids (Hole and Williams, 2020). Further investigation is needed to establish which other kinase pathways may have been affected by EC intervention in this study.

Alternatively, EC could have inhibited tau phosphorylation and oligomerization by binding to tau directly. GSPE which contains EC and other flavan-3-ols has been shown expand the width of PHFs from AD patients and reduce antibody labeling at several phosphoepitopes including AT8 and Ser262 (12E8; Ksiezak-Reding et al., 2012). However, EC itself was not able to inhibit heparin-induced tau aggregation *in vitro* (George et al., 2013) and it is more likely that the observed effects with GSPE were due to the known anti-amyloidogenic actions of EGCG (Wobst et al., 2015). While there are several intraneuronal mechanisms through which EC could be affecting tau phosphorylation, the possibility that the effects seen were a result of modulation of the extra-neuronal environment cannot be ignored. EC is known to improve vascular function, and this has been shown to improve cognition in both mice and humans (van Praag et al., 2007; Bernatova, 2018; Haskell-Ramsay et al., 2018). Improved vascular function could potentially promote clearance of pathological tau species as well as boosting neuronal function to ameliorate tau phosphorylation and aggregation.

A consideration that is important for future mechanistic studies is that EC itself may not be the bioactive constituent. The EC metabolome has been characterized in both humans and rats and it is now known that EC is metabolized to at least 20 metabolites including structurally-related metabolites (GI tract metabolism) and valerolactones (microbiota metabolism; Borges et al., 2016, 2018; Ottaviani et al., 2016). Chronic supplementation of EC to rats resulted in higher concentrations of structurally-related metabolites in the brain than EC (valerolactones were not analyzed; Wang et al., 2012). Therefore, it is possible that metabolites of EC may be responsible for the changes in tau phosphorylation observed in this study rather than the native aglycone which could, therefore, be considered as acting as a pro-drug supplement. Indeed, the levels of EC administered to mice in this study could not be achieved in humans through dietary consumption alone and should not therefore, be extrapolated to form any dietary dose recommendations for dementia. Dietary flavan-3-ols have received increasing interest recently, with clinical trials underway to determine the effects of EGCG on cognitive decline in carriers of the AD risk gene APOE4 (PENSA; NCT03978052) and cocoa flavonoid intervention on cognitive decline in the aging population (COSMOS-Mind study; NCT03035201).

This study showed that short term, oral intervention with a high dose of EC inhibited tau phosphorylation and appears to have reduced the levels of potentially toxic oligomeric tau species. While the mechanisms underpinning the observed affect remain unclear and validation in additional tau mouse models and ultimately humans is required, the findings presented here further add to the existing evidence that EC has promise as a multi-modal AD therapeutic potentially as a supplement to a flavanol-3-ol rich diet.

## Data Availability Statement

The raw data supporting the conclusions of this article will be made available by the authors, without undue reservation.

## Ethics Statement

All procedures were carried out in accordance with the United Kingdom Animal (Scientific Procedures) Act 1986 and were approved by the Universities of Exeter (PPL P29FAC36A) and Bath Animal Welfare and Ethical Review Body.

## Author Contributions

LS, JB, and RW conceived and designed the study. KH and RW analyzed the data and wrote the manuscript. LS performed the *in vivo* intervention. GM and RW processed the tissue and performed biochemical analysis. KH performed the *in vitro* and GSK3β work. KH, JB, JM, and RW interpreted the findings and edited the manuscript. RW, JB, and JM supervised the project. All authors read and approved the final manuscript.

## Conflict of Interest

RW has received an unrestricted grant from Mars Inc. The remaining authors declare that the research was conducted in the absence of any commercial or financial relationships that could be construed as a potential conflict of interest.
